# 4-Eth­oxy­benzohydrazide

**DOI:** 10.1107/S1600536812038998

**Published:** 2012-09-19

**Authors:** Muhammad Farman, Saira Khanum, Shahid Hameed, Peter G. Jones, Tanveer Ahmad

**Affiliations:** a Department of Chemistry, Quaid-i-Azam University, Islamabad 45320, Pakistan; b Institut für Anorganische und Analytische Chemie, Technische Universität Braunschweig, Postfach 3329, 38023 Braunschweig, Germany

## Abstract

The title compound, C_9_H_12_N_2_O_2_, is approximately planar (r.m.s. deviation = 0.13 Å for all non-H atoms). The carbonyl O atom is involved as acceptor in three different hydrogen-bond inter­actions. One N—H⋯O and the C—H⋯O(carbonyl) contact together with a weak C—H⋯O(eth­oxy) interaction link the mol­ecules into sheets parallel to (102). These are further linked into a three-dimensional network *via* the remaining C—H⋯O(carbon­yl) hydrogen bond and a C(methyl­ene)—H⋯π inter­action

## Related literature

For the meth­oxy analogue of the title compound, see: Ashiq *et al.* (2009 ▶). For biological properties of hydrazides, see: Gohil *et al.* (2010 ▶); Bordoloi *et al.* (2009 ▶); Kumar *et al.* (2009 ▶). For the use of hydrazides as precursors for the syntheses of heterocyclic compounds, see: Akhtar *et al.* (2010 ▶); Akhtar, Hameed, Al-Masoudi *et al.* (2008 ▶); Akhtar, Hameed, Khan *et al.* (2008 ▶); Khan, Akhtar *et al.* (2010 ▶); Khan, Hameed *et al.* (2010 ▶); Serwar *et al.* (2009 ▶); Syed *et al.* (2011 ▶); Zahid *et al.* (2009 ▶); Zia *et al.* (2012 ▶). For a description of the Cambridge Structural Database, see: Allen (2002 ▶); For details of the preparation, see: Furniss *et al.* (1989 ▶).
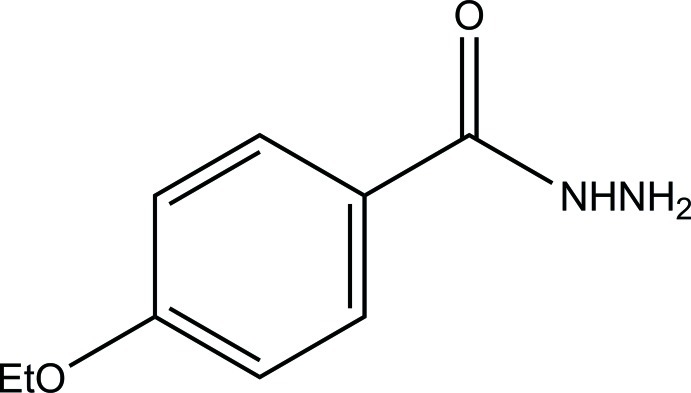


## Experimental

### 

#### Crystal data

C_9_H_12_N_2_O_2_
*M* *_r_* = 180.21
Monoclinic, 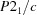

*a* = 10.8848 (3) Å
*b* = 10.0453 (2) Å
*c* = 8.4420 (3) Å
β = 110.669 (4)°
*V* = 863.64 (4) Å^3^
*Z* = 4
Mo *K*α radiation
μ = 0.10 mm^−1^
*T* = 100 K
0.3 × 0.2 × 0.2 mm

#### Data collection

Oxford Diffraction Xcalibur Eos diffractometer
42766 measured reflections
2874 independent reflections
2478 reflections with *I* > 2σ(*I*)
*R*_int_ = 0.024

#### Refinement

*R*[*F* ^2^ > 2σ(*F* ^2^)] = 0.034
*wR*(*F* ^2^) = 0.106
*S* = 1.10
2874 reflections
131 parameters
H atoms treated by a mixture of independent and constrained refinement
Δρ_max_ = 0.48 e Å^−3^
Δρ_min_ = −0.22 e Å^−3^

### 

Data collection: *CrysAlis PRO* (Oxford Diffraction, 2009 ▶); cell refinement: *CrysAlis PRO*; data reduction: *CrysAlis PRO*; program(s) used to solve structure: *SHELXS97* (Sheldrick, 2008 ▶); program(s) used to refine structure: *SHELXL97* (Sheldrick, 2008 ▶); molecular graphics: *XP* in *SHELXTL* (Sheldrick, 2008 ▶); software used to prepare material for publication: *SHELXL97*.

## Supplementary Material

Crystal structure: contains datablock(s) I, global. DOI: 10.1107/S1600536812038998/lr2080sup1.cif

Structure factors: contains datablock(s) I. DOI: 10.1107/S1600536812038998/lr2080Isup2.hkl

Supplementary material file. DOI: 10.1107/S1600536812038998/lr2080Isup3.cml

Additional supplementary materials:  crystallographic information; 3D view; checkCIF report

## Figures and Tables

**Table 1:** Hydrogen-bond geometry (Å, °) *Cg* is the centroid of the C1–C6 benzene ring

*D*—H⋯*A*	*D*—H	H⋯*A*	*D*⋯*A*	*D*—H⋯*A*
N2—H03⋯O1^i^	0.865 (13)	2.083 (13)	2.9290 (9)	165.6 (11)
C6—H6⋯O1^i^	0.95	2.39	3.3149 (9)	165
C3—H3⋯O2^ii^	0.95	2.61	3.5428 (9)	168
N1—H01⋯O1^iii^	0.933 (13)	2.212 (14)	3.1207 (9)	164.1 (12)
C8—H8*B*⋯*Cg* ^iv^	0.99	2.65	3.499 (1)	145

Symmetry codes: (i) 
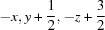
; (ii) 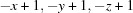; (iii) 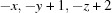; (iv) 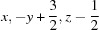.

**Fig. 1.**
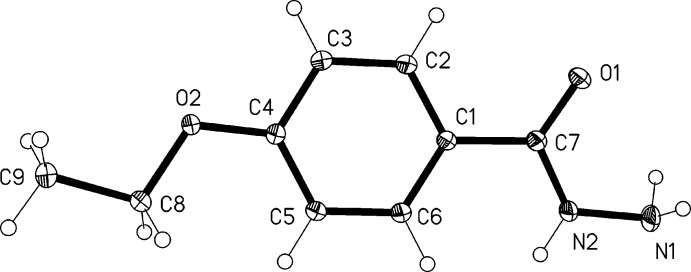
Molecular structure of the title compound. Ellipsoids represent 50% probability levels.

**Fig. 2.**
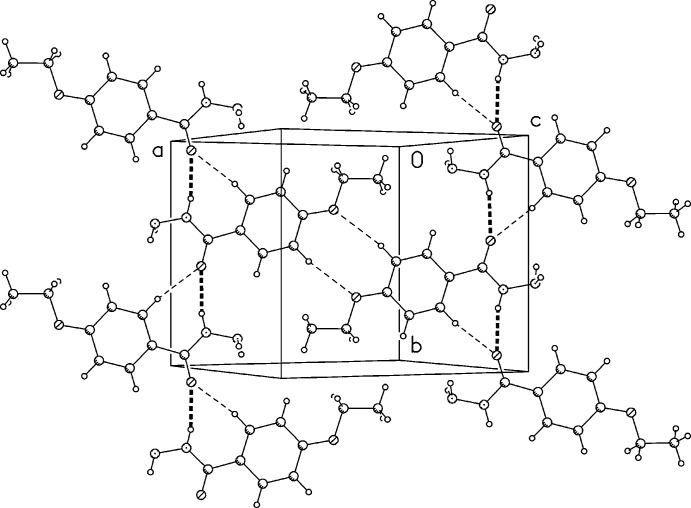
A view of the packing scheme, showing the layers parallel to (102). Thick dashed bonds represent classical H bonds and thin dashed bonds represent weak hydrogen bonds.

## supplementary crystallographic information

### Comment

Hydrazides represent one of the most biologically active classes of compounds reported in the chemical literature; they display a wide variety of biological activities such as antimicrobial (Kumar *et al.*, 2009) anticancer (Gohil *et al.*, 2010) and antigenotoxic (Bordoloi *et al.*, 2009). They have been employed as synthetic precursors for a number of hetero-cyclic compounds such as oxadiazoles, triazoles and thiadiazoles (Zia *et al.*, 2012; Syed *et al.*, 2011; Akhtar *et al.*, 2010; Akhtar, Hameed, Al-Masoudi *et al.*, 2008; Akhtar, Hameed, Khan *et al.*, 2008; Khan, Akhtar *et al.* , 2010; Khan, Hameed *et al.* , 2010; Serwar *et al.*, 2009; Zahid *et al.*, 2009). The title compound (1) was synthesized as an intermediate for its subsequent conversion to 1,2,4-triazoles and 1,3,4-thiadiazoles in order to explore their potential as antibacterial or antifungal agents or urease inhibitors.

The structure of (1) is shown in Fig. 1. Molecular dimensions may be regarded as normal, *e.g.* the N—N bond length of 1.4117 (9) Å; a search of the Cambridge Structural Database (CSD, *CONQUEST* Version 1.14; Allen, 2002) for the benzohydrazine fragment gave 37 hits (41 molecules) with an average N—N bond length of 1.415 (5) Å. The molecule is approximately planar, with an r.m.s. deviation of 0.13 Å for all non-H atoms. The angle between the phenyl and CON_2_ planes is 14.65 (6)°. The hydrogen atoms of the NH_2_ group lie to either side of the CON_2_ plane, with torsion angles C7—N2—N1—H01 61.8 (9)° and C7—N2—N1—H02 - 53.1 (8)°.

The carbonyl oxygen is involved as acceptor in three different hydrogen bond interactions. Two of them form a bifurcated N2—H03···O1^(i) ^, C6—H6···O1^(i) ^system, these interactions together with a very weak C3—H3···O2^(ii)^ (ethoxy) hydrogen bond link the molecules into sheets parallel to (102). These layers are further linked into a three-dimensional network *via* the remaining N1—H01···O1^(iii)^ (carbonyl) hydrogen bond and a C8—H8B···Cg^(iv)^π interaction, where Cg is the centroid of the C1-C6 benzene ring [symmetry codes: (i) -x, y+1/2,-z+3/2;(ii) -x+1,-y+1,-z+1; (iii)-x, -y+1,-z+2 and (iv) x, -y+3/2, z-1/2]. The hydrogen H02 is not involved in hydrogen bonding interactions.

Compound (1) is not isotypic to its methoxy analogue (Ashiq *et al.*, 2009), which crystallizes in *P*2_1_2_1_2_1_.

### Experimental

3.6 g of methyl *p*-ethoxybenzoate was added to 40 ml freshly distilled methanol in a round-bottomed flask. The content was stirred until completely dissolved and the flask was fitted with a reflux condenser bearing a calcium chloride guard tube. Then 2.0 g of 80% hydrazine hydrate was added slowly. The reaction was monitored by thin layer chromatography. Upon completion of the reaction, the content was concentrated *in vacuo* (Furniss *et al.*, 1989). The resulting crude solid was filtered, washed with water and agitated with freshly distilled acetone for 1 h. The product was then recrystallized from aqueous ethanol.

### Refinement

The NH hydrogen atoms were refined freely. Methyl H atoms were identified in difference syntheses, idealized and refined corresponding to a rigid group with C—H 0.98 Å and H—C—H angles 109.5°, allowed to rotate but not tip. Other H atoms were placed in calculated positions and refined using a riding model with C—H_arom_= 0.95 and *C*—H_methylene_ =0.99 Å; the hydrogen *U* values were fixed at 1.5 (methyl) or 1.2 × *U*(eq) of the parent atom.

### Crystal data

**Table:**

C_9_H_12_N_2_O_2_	*F*(000) = 384
*M**_r_* = 180.21	*D*_x_ = 1.386 Mg m^−^^3^
Monoclinic, *P*2_1_/*c*	Melting point: 403 K
Hall symbol: -P 2ybc	Mo *K*α radiation, λ = 0.71073 Å
*a* = 10.8848 (3) Å	Cell parameters from 23790 reflections
*b* = 10.0453 (2) Å	θ = 2.6–32.6°
*c* = 8.4420 (3) Å	µ = 0.10 mm^−^^1^
β = 110.669 (4)°	*T* = 100 K
*V* = 863.64 (4) Å^3^	Block, colourless
*Z* = 4	0.3 × 0.2 × 0.2 mm

### Data collection

**Table:**

Oxford Diffraction Xcalibur Eos diffractometer	2478 reflections with *I* > 2σ(*I*)
Radiation source: Enhance (Mo) X-ray Source	*R*_int_ = 0.024
Graphite monochromator	θ_max_ = 31.5°, θ_min_ = 2.9°
Detector resolution: 16.1419 pixels mm^-1^	*h* = −15→15
ω scan	*k* = −14→14
42766 measured reflections	*l* = −12→12
2874 independent reflections	

### Refinement

**Table:**

Refinement on *F*^2^	Primary atom site location: structure-invariant direct methods
Least-squares matrix: full	Secondary atom site location: difference Fourier map
*R*[*F*^2^ > 2σ(*F*^2^)] = 0.034	Hydrogen site location: inferred from neighbouring sites
*wR*(*F*^2^) = 0.106	H atoms treated by a mixture of independent and constrained refinement
*S* = 1.10	*w* = 1/[σ^2^(*F*_o_^2^) + (0.0734*P*)^2^ + 0.0351*P*] where *P* = (*F*_o_^2^ + 2*F*_c_^2^)/3
2874 reflections	(Δ/σ)_max_ = 0.002
131 parameters	Δρ_max_ = 0.48 e Å^−^^3^
0 restraints	Δρ_min_ = −0.22 e Å^−^^3^

### Special details

**Table:**

Geometry. All e.s.d.'s (except the e.s.d. in the dihedral angle between two l.s. planes) are estimated using the full covariance matrix. The cell e.s.d.'s are taken into account individually in the estimation of e.s.d.'s in distances, angles and torsion angles; correlations between e.s.d.'s in cell parameters are only used when they are defined by crystal symmetry. An approximate (isotropic) treatment of cell e.s.d.'s is used for estimating e.s.d.'s involving l.s. planes.Least-squares planes (*x*,*y*,*z* in crystal coordinates) and deviations from them (* indicates atom used to define plane)3.5814 (0.0029) *x* - 0.3102 (0.0031) *y* + 6.4744 (0.0016) *z* = 4.7086 (0.0021)* 0.0107 (0.0005) C1 * -0.0025 (0.0005) C2 * -0.0096 (0.0005) C3 * 0.0135 (0.0005) C4 * -0.0052 (0.0005) C5 * -0.0070 (0.0005) C6 0.1090 (0.0011) C7 0.1528 (0.0013) C8 0.3370 (0.0017) C9 0.4145 (0.0012) O1 0.0757 (0.0010) O2 - 0.0498 (0.0016) N1 - 0.1309 (0.0013) N2Rms deviation of fitted atoms = 0.00893.5873 (0.0049) *x* + 2.2383 (0.0045) *y* + 6.2646 (0.0032) *z* = 6.0909 (0.0020)Angle to previous plane (with approximate e.s.d.) = 14.65 (0.06)* 0.0002 (0.0004) C7 * -0.0001 (0.0002) O1 * 0.0001 (0.0002) N1 * -0.0002 (0.0004) N2Rms deviation of fitted atoms = 0.0001
Refinement. Refinement of *F*^2^ against ALL reflections. The weighted *R*-factor *wR* and goodness of fit *S* are based on *F*^2^, conventional *R*-factors *R* are based on *F*, with *F* set to zero for negative *F*^2^. The threshold expression of *F*^2^ > σ(*F*^2^) is used only for calculating *R*-factors(gt) *etc*. and is not relevant to the choice of reflections for refinement. *R*-factors based on *F*^2^ are statistically about twice as large as those based on *F*, and *R*- factors based on ALL data will be even larger.

### Fractional atomic coordinates and isotropic or equivalent isotropic displacement parameters (Å^2^)

**Table:**

	*x*	*y*	*z*	*U*_iso_*/*U*_eq_	
C1	0.14875 (7)	0.60114 (7)	0.67544 (8)	0.01178 (14)	
C2	0.21991 (7)	0.50128 (7)	0.62925 (9)	0.01315 (14)	
H2	0.1982	0.4104	0.6363	0.016*	
C3	0.32166 (7)	0.53422 (7)	0.57344 (9)	0.01377 (14)	
H3	0.3685	0.4660	0.5411	0.017*	
C4	0.35534 (7)	0.66779 (7)	0.56479 (9)	0.01238 (14)	
C5	0.28323 (7)	0.76797 (7)	0.60658 (9)	0.01476 (15)	
H5	0.3040	0.8589	0.5977	0.018*	
C6	0.18082 (7)	0.73392 (7)	0.66132 (9)	0.01406 (15)	
H6	0.1319	0.8023	0.6896	0.017*	
C7	0.04616 (7)	0.56096 (7)	0.74544 (9)	0.01238 (14)	
C8	0.50091 (7)	0.82661 (7)	0.51338 (10)	0.01446 (15)	
H8A	0.5170	0.8694	0.6245	0.017*	
H8B	0.4315	0.8771	0.4259	0.017*	
C9	0.62551 (8)	0.82519 (8)	0.47285 (10)	0.01797 (16)	
H9A	0.6925	0.7725	0.5582	0.027*	
H9B	0.6572	0.9165	0.4733	0.027*	
H9C	0.6076	0.7856	0.3608	0.027*	
N1	−0.13631 (7)	0.63025 (7)	0.82516 (9)	0.01874 (15)	
H01	−0.0919 (13)	0.6029 (13)	0.9364 (17)	0.039 (3)*	
H02	−0.1806 (12)	0.5584 (13)	0.7605 (16)	0.033 (3)*	
N2	−0.03653 (6)	0.65623 (6)	0.75869 (8)	0.01518 (14)	
H03	−0.0305 (11)	0.7382 (13)	0.7308 (14)	0.026 (3)*	
O1	0.03801 (5)	0.44481 (5)	0.79156 (7)	0.01735 (13)	
O2	0.46083 (5)	0.69025 (5)	0.51711 (7)	0.01480 (13)	

### Atomic displacement parameters (Å^2^)

**Table:**

	*U*^11^	*U*^22^	*U*^33^	*U*^12^	*U*^13^	*U*^23^
C1	0.0120 (3)	0.0097 (3)	0.0148 (3)	0.0001 (2)	0.0061 (2)	0.0006 (2)
C2	0.0147 (3)	0.0096 (3)	0.0165 (3)	−0.0004 (2)	0.0072 (2)	−0.0006 (2)
C3	0.0156 (3)	0.0104 (3)	0.0175 (3)	0.0008 (2)	0.0086 (3)	−0.0006 (2)
C4	0.0129 (3)	0.0112 (3)	0.0149 (3)	0.0005 (2)	0.0073 (2)	0.0006 (2)
C5	0.0167 (3)	0.0095 (3)	0.0216 (3)	0.0006 (2)	0.0112 (3)	0.0012 (2)
C6	0.0151 (3)	0.0103 (3)	0.0198 (3)	0.0012 (2)	0.0100 (3)	0.0008 (2)
C7	0.0122 (3)	0.0109 (3)	0.0147 (3)	−0.0008 (2)	0.0057 (2)	−0.0002 (2)
C8	0.0163 (3)	0.0102 (3)	0.0197 (3)	−0.0009 (2)	0.0099 (3)	0.0001 (2)
C9	0.0170 (3)	0.0150 (3)	0.0261 (4)	−0.0019 (2)	0.0128 (3)	−0.0007 (3)
N1	0.0168 (3)	0.0200 (3)	0.0247 (3)	0.0005 (2)	0.0139 (3)	0.0032 (3)
N2	0.0152 (3)	0.0117 (3)	0.0232 (3)	0.0008 (2)	0.0125 (2)	0.0025 (2)
O1	0.0196 (3)	0.0106 (3)	0.0260 (3)	0.00006 (19)	0.0133 (2)	0.0031 (2)
O2	0.0159 (3)	0.0105 (2)	0.0229 (3)	−0.00056 (18)	0.0128 (2)	0.00054 (19)

### Geometric parameters (Å, º)

**Table:**

C1—C6	1.3944 (10)	C2—H2	0.9500
C1—C2	1.4041 (10)	C3—H3	0.9500
C1—C7	1.4916 (10)	C5—H5	0.9500
C2—C3	1.3879 (10)	C6—H6	0.9500
C3—C4	1.3997 (10)	C8—H8A	0.9900
C4—O2	1.3630 (8)	C8—H8B	0.9900
C4—C5	1.3959 (10)	C9—H9A	0.9800
C5—C6	1.3917 (10)	C9—H9B	0.9800
C7—O1	1.2431 (8)	C9—H9C	0.9800
C7—N2	1.3452 (9)	N1—H01	0.933 (13)
C8—O2	1.4413 (9)	N1—H02	0.931 (13)
C8—C9	1.5114 (10)	N2—H03	0.865 (13)
N1—N2	1.4117 (9)		
			
C6—C1—C2	118.70 (6)	C6—C5—H5	120.2
C6—C1—C7	122.53 (6)	C4—C5—H5	120.2
C2—C1—C7	118.70 (6)	C5—C6—H6	119.4
C3—C2—C1	120.56 (6)	C1—C6—H6	119.4
C2—C3—C4	120.09 (6)	O2—C8—H8A	110.2
O2—C4—C5	124.25 (6)	C9—C8—H8A	110.2
O2—C4—C3	115.95 (6)	O2—C8—H8B	110.2
C5—C4—C3	119.79 (6)	C9—C8—H8B	110.2
C6—C5—C4	119.63 (7)	H8A—C8—H8B	108.5
C5—C6—C1	121.17 (6)	C8—C9—H9A	109.5
O1—C7—N2	121.19 (6)	C8—C9—H9B	109.5
O1—C7—C1	121.62 (6)	H9A—C9—H9B	109.5
N2—C7—C1	117.19 (6)	C8—C9—H9C	109.5
O2—C8—C9	107.38 (6)	H9A—C9—H9C	109.5
C7—N2—N1	122.19 (6)	H9B—C9—H9C	109.5
C4—O2—C8	117.18 (5)	N2—N1—H01	104.9 (8)
C3—C2—H2	119.7	N2—N1—H02	102.9 (7)
C1—C2—H2	119.7	H01—N1—H02	109.8 (11)
C2—C3—H3	120.0	C7—N2—H03	122.5 (8)
C4—C3—H3	120.0	N1—N2—H03	115.3 (8)
			
C6—C1—C2—C3	1.10 (10)	C6—C1—C7—O1	−164.06 (7)
C7—C1—C2—C3	−176.01 (6)	C2—C1—C7—O1	12.93 (10)
C1—C2—C3—C4	0.82 (11)	C6—C1—C7—N2	15.13 (10)
C2—C3—C4—O2	176.66 (6)	C2—C1—C7—N2	−167.88 (6)
C2—C3—C4—C5	−2.32 (11)	O1—C7—N2—N1	0.05 (11)
O2—C4—C5—C6	−177.01 (6)	C1—C7—N2—N1	−179.15 (6)
C3—C4—C5—C6	1.89 (11)	C5—C4—O2—C8	1.26 (10)
C4—C5—C6—C1	0.05 (11)	C3—C4—O2—C8	−177.68 (6)
C2—C1—C6—C5	−1.54 (11)	C9—C8—O2—C4	174.98 (6)
C7—C1—C6—C5	175.46 (6)		

### Hydrogen-bond geometry (Å, º)

Cg is the centroid of the C1–C6 benzene ring

**Table:**

*D*—H···*A*	*D*—H	H···*A*	*D*···*A*	*D*—H···*A*
N2—H03···O1^i^	0.865 (13)	2.083 (13)	2.9290 (9)	165.6 (11)
C6—H6···O1^i^	0.95	2.39	3.3149 (9)	165
C3—H3···O2^ii^	0.95	2.61	3.5428 (9)	168
N1—H01···O1^iii^	0.933 (13)	2.212 (14)	3.1207 (9)	164.1 (12)
C8—H8*B*···*Cg*^iv^	0.99	2.65	3.499 (1)	145

Symmetry codes: (i) −*x*, *y*+1/2, −*z*+3/2; (ii) −*x*+1, −*y*+1, −*z*+1; (iii) −*x*, −*y*+1, −*z*+2; (iv) *x*, −*y*+3/2, *z*−1/2.
